# Transorbital Brain Injury by a Sharp Object: A Case Report and Literature Review

**DOI:** 10.7759/cureus.93674

**Published:** 2025-10-01

**Authors:** Noé Pérez Carrillo, Arturo Larrazolo Lopez, Alejandro Mendez-Viveros, Naomi Contreras Galván, Manuel Angeles-Castellanos, Alejandra Minerva Méndez López, Amira Deneb Trejo Villalobos, Ricardo Carlos Sandoval Quiroa, Victor Eduardo Chao Rodríguez

**Affiliations:** 1 Department of Neurosurgery, Hospital General de México "Dr. Eduardo Liceaga", Mexico City, MEX; 2 Faculty of Medicine, Universidad Nacional Autónoma de México, Mexico City, MEX; 3 Anatomical Sciences, Faculty of Medicine, Universidad Nacional Autónoma de México, Mexico City, MEX; 4 Social Service, Universidad Tecnológica de Monterrey, Monterrey, MEX

**Keywords:** brain injury, case report, head trauma, sharp object, transorbital

## Abstract

Non-missile penetrating head injuries are rare in Western countries and often present significant management challenges. We describe the case of a 39-year-old man who sustained a transorbital penetrating brain injury after an assault with a sharp object. On admission, he presented with ocular trauma and impaired consciousness. Computed tomography revealed a metallic object entering through the right orbit, traversing to the left parasagittal occipital bone, with associated intraventricular and subarachnoid hemorrhage. The foreign body was surgically removed without intraoperative complications, followed by ocular enucleation and dural repair. His condition gradually improved, and he was discharged with a Glasgow Coma Scale score of 11. At the one-month follow-up, he demonstrated preserved lower limb strength with mild residual left upper limb paresis, and is currently under rehabilitation. This case highlights the rarity and complexity of orbitocranial penetrating injuries, underscoring the importance of timely diagnosis, careful surgical planning, and multidisciplinary management to optimize functional outcomes.

## Introduction

Non-missile penetrating head injuries (NMPHIs) are exceptionally rare in Western countries but are associated with high morbidity and mortality. These injuries pose unique diagnostic and therapeutic challenges due to their complexity and the limited number of reported cases. NMPHIs are defined as brain injuries caused by a sharp object driven through the skull, producing focal damage along its tract without the thermal or kinetic effects typical of high-velocity projectiles [1]. Knives are the most frequent causative objects, with the orbit representing the most common entry site (86%) because of its anatomical vulnerability [2-4]. Although accidental mechanisms predominate worldwide, in regions with high rates of interpersonal violence, such as South Africa, assault is the leading cause [1]. Stab wounds to the brain account for only about 0.4% of head injuries in Western populations [2]; transorbital penetrating injuries remain exceptionally uncommon, and the literature is therefore limited. Despite their severity, there are no standardized guidelines for their management, and reports from Latin America are particularly scarce. We present the case of a 39-year-old man who survived a transorbital brain injury caused by a sharp object, highlighting the surgical strategy and favorable functional recovery.

## Case presentation

A 39-year-old man with no relevant medical history was initially admitted to a private hospital after sustaining ocular trauma from an assault with a sharp object. Due to the lack of qualified personnel, he was referred to our institution for specialized care. On arrival, the patient was sedated with propofol and midazolam and received analgesia with buprenorphine. He was orotracheally intubated, on assisted ventilation with a tidal volume of 450 mL and positive end-expiratory pressure of 5, without vasopressor support. Neurologically, he presented with a Richmond Agitation-Sedation Scale (RASS) score of -5 and a left pinpoint pupil; brainstem reflexes were not assessable, and other neurological assessments were limited due to sedation.

**Figure 1:**
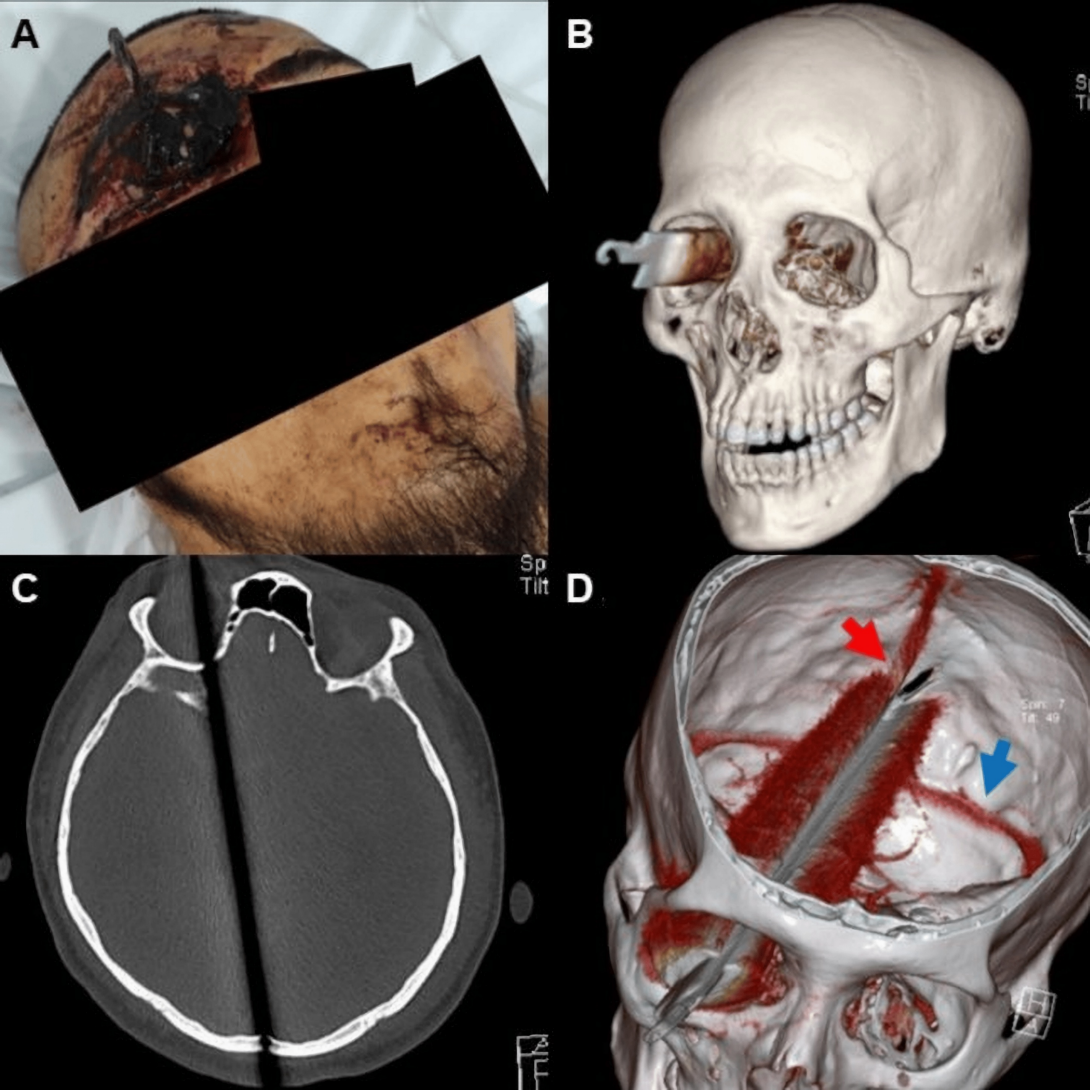
(A) Physical examination of the transorbital trauma. (B) Computer tomography (CT) 3D preoperative reconstruction where the entry of the cutting object into the orbit is appreciable. (C) Bone window of the CT where the trajectory of the knife from the right orbit to the left parasagittal line, passing through the entire occipital bone, is observed. (D) CT 3D reconstruction where it is observed that the sharp object passes too close to the superior sagittal sinus (red arrow) and the transverse sinus (blue arrow).

Cranial computer tomography (CT) performed at our institution revealed a laminar hyperdense metallic object with intense artifact, entering through the right orbit via the globe, distorting orbital anatomy. The object passed through the superior orbital fissure and greater wing of the sphenoid toward the cranial cavity, fragmenting the floor of the anterior cranial fossa and the lateral clinoid process, and impacting the lateral eminence of the occipital cruciform eminence, contacting the outer table (Figure 1). Blood was detected in both occipital horns and the fourth ventricle, accompanied by a Greene II subarachnoid hemorrhage. Laminar subarachnoid hematomas were also noted in the parietal gyri, interhemispheric surface, dorsocaudal cerebellar tentorium, and prepontine cisterns.

**Figure 2:**
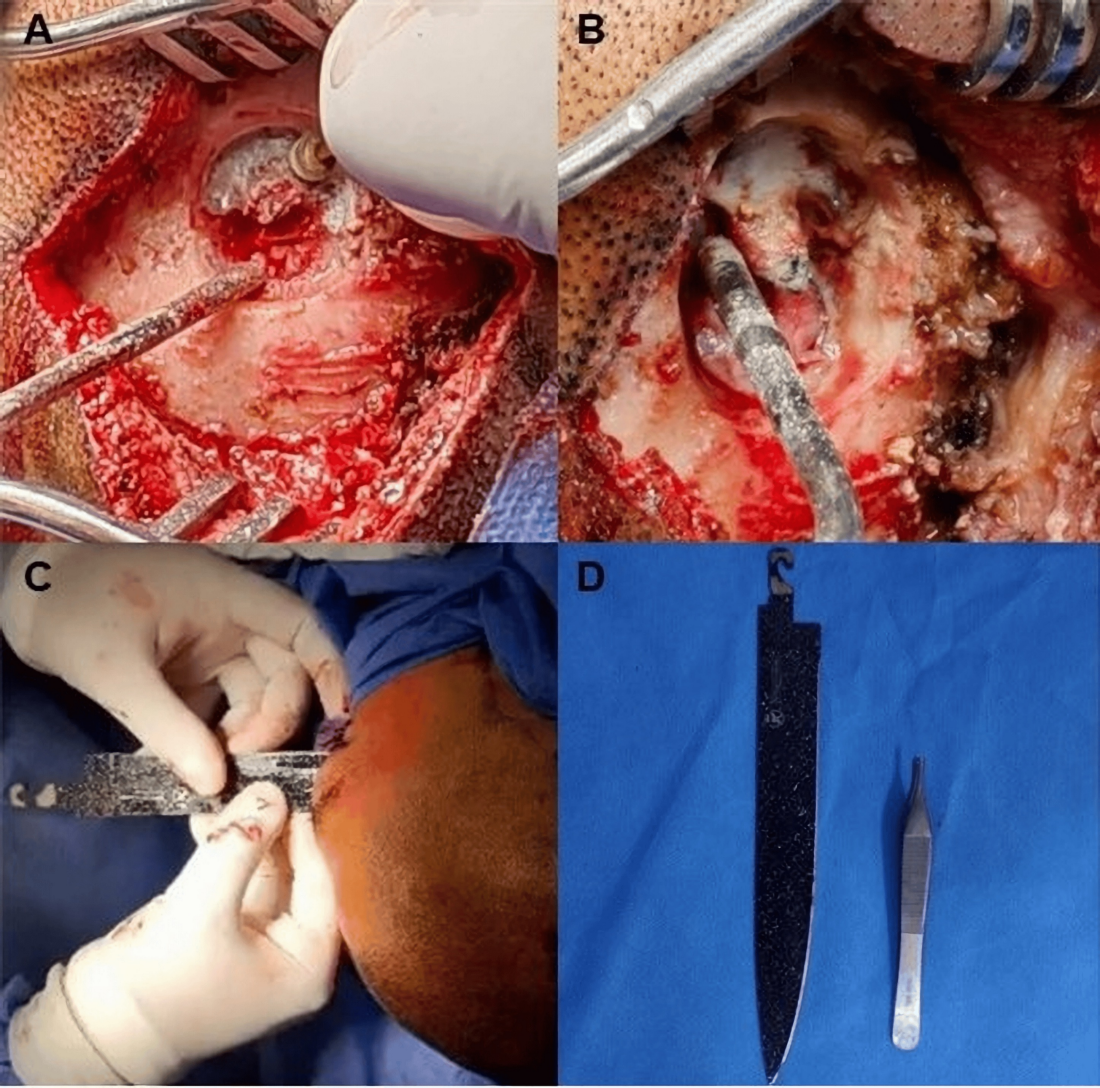
(A) Drilling around the exit point of the knife tip to free it. (B) The inner table is visible and thinner. (C) Delicate traction is performed with monitoring. (D) The entire sharp object is removed, with its size compared with common dissecting forceps.

The ophthalmology team performed enucleation of the right globe due to traumatic disruption. For the neurosurgical procedure, the patient was positioned supine with left cephalic rotation under balanced general anesthesia. Considering the trajectory of the foreign body from the right orbit to the occipital bone and its proximity to the sagittal sinus, a longitudinal occipital midline incision was chosen to allow direct visualization of the object’s tip, safe exposure of the dural surface, and controlled extraction, minimizing the risk of vascular injury and intracranial hemorrhage. After asepsis, antisepsis, and placement of sterile drapes, the incision was made. The surrounding bone was carefully drilled using a number 5 diamond burr to adequately expose the tip of the object while preserving the sagittal sinus and intact dura. The object was removed in its entirety. Intraoperative findings included tense cerebral tissue and material with purulent characteristics, without an identifiable exit site (Figure 2). Upon dural incision, abundant cerebrospinal fluid was released without the purulent content. Hemostasis was achieved using Gelfoam and Tisseel, and the wound was closed in layers with Vicryl and Prolene, with placement of a subgaleal drain.

**Figure 3:**
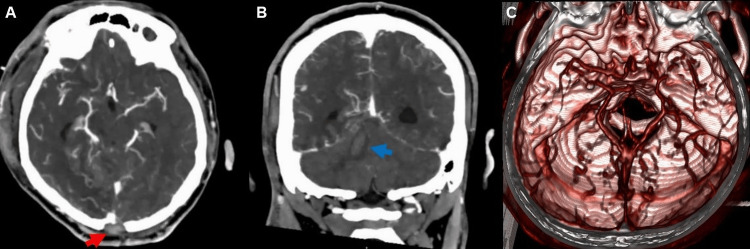
(A) Postoperative axial contrast CT shows changes in the occipital bone (red arrow), and absence of hemorrhage is noted. (B) Edema is found along the trajectory of the knife (blue arrow). (C) 3D vascular reconstruction showing no evidence of injury.

The surgical procedure was concluded, and postoperative imaging was obtained (Figure 3).

A second procedure involved primary dural repair via pterional craniotomy, ensuring no cerebrospinal fluid leak or vascular compromise.

The patient was initially admitted to the intensive care unit and later transferred to the general ward following improvement. He was eventually discharged from the hospital with a Glasgow Coma Scale score of 11, limited primarily by verbal response but without major alterations. The total hospitalization lasted about three weeks, after which discharge was authorized due to satisfactory clinical progress and the absence of systemic inflammatory response. At the follow-up outpatient consultation, the patient showed paresis predominantly in the left upper limb; physical rehabilitation is continued.

At present, almost 23 months after surgery, he remains alive and stable, without substantial changes when compared to his immediate postoperative course.

## Discussion

**Figure 4:**
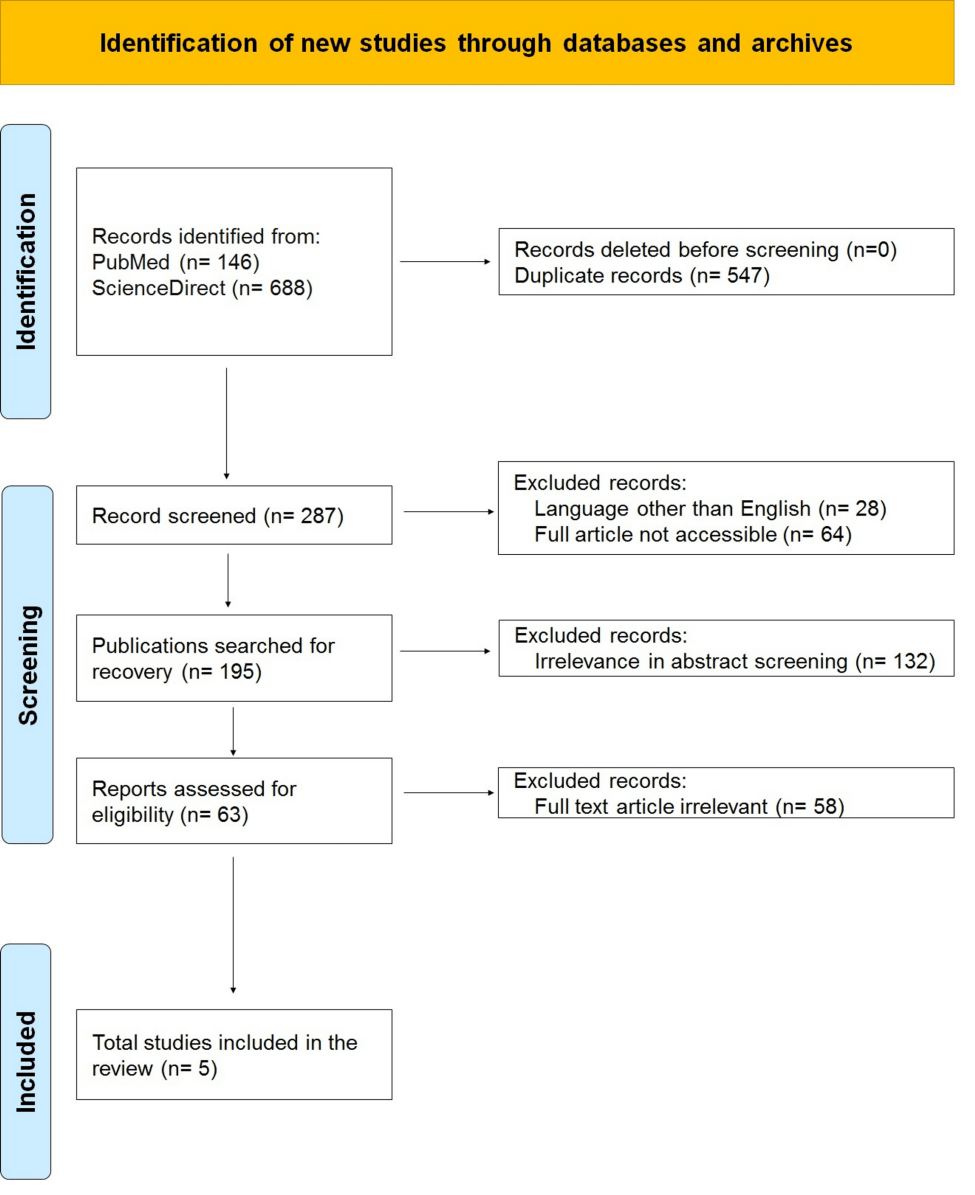
PRISMA flowchart for selecting the cases PRISMA: Preferred Reporting Items for Systematic Reviews and Meta-Analyses

To contextualize this case, we conducted a literature review to summarize current evidence on transorbital non-missile penetrating brain injuries. PubMed and ScienceDirect were searched using the terms “transorbital brain injury”, “non-missile penetrating head trauma”, “NMPHIs”, and “low-velocity orbitocranial trauma”. Inclusion criteria included case reports and series published in English from 2000 to 2023, reporting clinical, radiological, and surgical outcomes. Exclusion criteria encompassed missile injuries, pediatric patients, and non-orbitocranial penetrating trauma (Figure 4).

Penetrating cranial trauma is considerably less common than blunt cranial trauma, with a reported incidence of approximately 0.4% [5]. Orbitocranial injuries caused by low-velocity objects, such as the one seen in our patient, are even rarer. Only a limited number of transorbital brain injury cases have been documented in the literature. Despite their low incidence, these injuries can result in significant ophthalmological and neurological complications.

The standard orbital depth is roughly 5 cm, meaning that objects exceeding this length can readily reach intracranial structures. Typically, penetration occurs through the orbital roof due to the thin frontal bone. In our patient, the object traversed the superior orbital fissure, ultimately contacting the medial temporal lobe and lateral aspect of the brainstem before impacting the occipital bone in the contralateral parasagittal line. Remarkably, despite this deep trajectory, the patient sustained only mild long-term deficits, likely due to the small diameter of the object, avoidance of major vascular structures, and prompt surgical intervention.

The reported mortality for transorbital penetrating injuries varies: Liu et al. (2011) described approximately 25% mortality [2], whereas Etawi et al. (2023) reported a 12% mortality rate [4]. Given the high morbidity and mortality associated with these injuries, standardized management protocols have been proposed. Initial guidelines for penetrating brain injuries were published in 2001 but remain largely outdated. Schreckinger et al. (2011) proposed a management algorithm [6], which we compared with our therapeutic approach and adapted to the specifics of this case (Table 1).

**Table 1:** Reported cases of NMPHIs in the literature NMPHIs: non-missile penetrating head injuries

Author	Year	Number of patients	Patients with transorbital brain injury	Age	Sex	Entry way	Object	Intracranial haemorrhage	Pre-hospital management	Emergency management	Surgical intervention	Post-hospital management	Complications
Chowdhury et al. [7]	2016	17	5	53	Male	Left supraorbital	Polyspike teta	No	Not reported	Not reported	Drilling around the penetrated spike, followed by a small craniotomy	Not reported	None
				40	Female	Supraorbital frontal bone	A stone with a blunt apex projecting in an upward direction	No	Partially stitched injury	Not reported	Right pterional craniotomy, removal of frontal hematoma, decompression of the superior orbital fissure and optic canal, surgical toileting, elevation of bone fragments, and reconstruction of the orbital rim	Not reported	Right-sided vision loss
				11	Male	Right orbit	Long bamboo stick	No	Not reported	Not reported	Right-sided evisceration and burr hole aspiration of brain abscess with aggressive antibiotic therapy	Not reported	One-sided orbital cellulitis, vision loss, and brain abscess
				45	Male	Left orbit	Long, sharp bamboo stick	No	Not reported	Not reported	Transpalpabral removal	Not reported	Vision loss in left eye
				23	Male	Right-sided frontal above the supraorbital ridge (paramedian) and frontal lobe	Bamboo stick	No	Not reported	Not reported	Small craniotomy	Not reported	None
Kitakami et al. [8]	1999	1	1	4	Male	Left orbit	Knife	No	Sedation	Antibiotics	Pterional, subtemporal, and lateral suboccipital combined approach; posterior fossa craniectomy	Not reported	Left-sided blindness, complete left ophthalmoplegia, and hypesthesia of the left upper face
Gulati et al. [9]	2010	1	1	33	Male	Right infra-orbital	Knife	No	Resuscitated according to the Advanced Trauma Life Support (ATLS) guidelines	Intubated	Bifrontal craniotomy, combined with a superior orbitotomy	Ophthalmological assessment and delayed internal cerebral artery angiogram 4 weeks following injury	Vision loss in the right eye; lost to follow-up
Okay et al. [10]	2009	1	1	42	Male	Medial epicanthus on the right eye	Knife	No	Not reported	Not reported	Pterional approach	Not reported	Total visual loss and absence of pupillary reflexes in the right eye
Schreckinger et al. [6]	2011	4	1	21	Male	Left and right orbit	Ink pen	Yes	Not reported	Sedation	Bilateral transorbital approach	The patient was transferred to the psychiatry service 2 weeks after his surgery.	Nonreactive to light bilaterally

In our case, the foreign body remained within the cranial cavity, justifying controlled removal in the operating room. Considering the heterogeneity of these lesions and the absence of updated protocols [1,6-10], we opted for a direct occipital approach tailored to the patient’s anatomy, which allowed safe exposure of the foreign body, protection of the sagittal sinus, and effective hemostatic control (Table 2). Potential complications, including hemorrhage, cerebrospinal fluid leak, infection, and damage to neurovascular structures, were carefully mitigated through meticulous operative planning and intraoperative monitoring.

**Table 2:** Therapeutic approach proposed Proposal by the authors of this work for future considerations of NMPHIs in highly specialized and trauma hospital settings.

Step	Intervention	Justification
1	Airway, breathing, circulation (ABC)	Prioritize life-saving measures, as in any trauma patient.
2	Object removal	Removing the penetrating object in an uncontrolled environment can lead to uncontrolled and fatal bleeding. Therefore, we first performed a simple cranial CT scan to assess the extent of intracranial involvement.
3	Vascular injury assessment	Given the object's nature, we proceeded to rule out vascular injury using angiography or angiotomography. Unfortunately, MRI was not feasible due to the object's composition.
4	Antibiotic therapy	We initiated antibiotic therapy with ceftriaxone and metronidazole.

This case illustrates that, even with deep intracranial penetration, favorable outcomes are possible if the object stays clear of major vascular structures and surgery is performed promptly. It emphasizes the urgent need for updated, evidence-based algorithms for the management of NMPHIs, particularly in low-incidence clinical settings.

## Conclusions

This case demonstrates that favorable outcomes are possible even after a deep transorbital brain injury when prompt, multidisciplinary, and individualized management is implemented. Due to the low incidence and heterogeneity of these injuries, there is no consensus on the optimal surgical approach, highlighting the need for case-specific planning. Clinicians facing similar injuries should prioritize careful imaging evaluation, controlled surgical removal of the foreign body, protection of critical neurovascular structures, and close perioperative monitoring. Early involvement of neurosurgery, ophthalmology, and critical care teams is essential. Systematic reporting and multicenter data collection are urgently needed to guide evidence-based protocols, optimize surgical strategies, and reduce morbidity and mortality in future cases.
